# Supplementary material for: Are carbon emissions trading and green financial instruments synergistic? -Comprehensive quantitative research based on content analysis
Source: PLoS One. 2024 Mar 7;19(3):e0298601. doi: 10.1371/journal.pone.0298601 (PMC10919601; doi:10.1371/journal.pone.0298601)
Supplement: S1 Data — (ZIP) [file pone.0298601.s001.zip › Data/地级市Z_F工作报告.zip!/╣π╢1⁄2/╢1⁄2▌╕╩╨/└┤╫╘╣1⁄2╓┌║┼í╛┬φ┐╦╩2╛▌═°í┐_2014.docx]

各位代表：

现在，我代表市人民政府，向大会报告2013年政府工作，对2014年工作提出建议，请予审议。并请政协各位委员和其他列席人员提出意见。

2013年工作回顾

刚刚过去的一年，是东莞加快推动高水平崛起的奋进之年。在市委的正确领导下，全市上下以党的十八大和十八届三中全会精神为指导，深入贯彻习近平总书记视察广东重要讲话精神，着力优环境、上项目、强统筹、抓改革，扎实推进各项工作，较好地完成了市第十五届人大三次会议确定的年度目标任务。

一年来，我们坚持稳中求进的总基调，实现了经济提速与转型加速。预计全市生产总值5500亿元，增长9.7%，快于全国全省平均水平，为“十二五”以来年度最高增速。来源于东莞的财政收入974亿元，增长15.2%，其中市公共财政预算收入409亿元，增长14.8%。进出口总额突破1500亿美元。各项人民币存款余额突破8000亿元，达到8595亿元。规模以上工业增加值、固定资产投资等实现近年最快增长。产业转型升级步伐加快，创新驱动力持续增强。东莞获得最具成长性创新型城市、国家知识产权示范城市等荣誉称号，成功成为第二批国家电子商务示范城市。

一年来，我们奏响工作落实年的主旋律，以实干抓出工作实效。发扬真抓实干的优良传统，狠抓市委各项决策的执行落实，狠抓“三重”建设的跟踪落实，狠抓十件民生实事、企业与基层减负、扶持镇村发展等任务的分解落实。部门镇街在争创“单打冠军”中实现了新的发展，东莞高水平崛起的基础进一步夯实。

一年来，我们凝聚改革创新的正能量，积极谋求重点领域突破。商事登记改革得到国家工商总局、省委省政府及广大企业的认可，行政审批事项精简一半以上，农村综合改革推动村组负债率降至近二十年最低，创新土地机制整合盘活土地近十万亩，节能减排获得中央财政大力支持，东莞成为承担全省创新社会管理试点工作最多的城市。一系列改革的锐意推进，为转型发展增创了制度红利。

一年来，我们厉行八项规定等新要求，在锤炼作风中提升政府形象。公务接待和庆典活动大幅压减，铺张浪费和奢靡之风有效遏制，文牍主义和形式主义切实改进，一批影响群众切身利益的问题得到及时解决，一批违法违纪典型案件得到严厉查处，深入基层、联系群众的氛围日渐形成，政府形象和社会满意度进一步提升。

一年来，我们主要抓了以下工作：

——狠抓市委和市政府1号文的贯彻落实。在全国率先构建提高开放型经济水平政策体系。成功举办加博会、漫博会、台博会，办展水平和档次有了明显提高。开展外贸九大课题调研，形成加工贸易产业提升综合服务体系等成果，为全国外经贸发展探索新路径。深化加工贸易审批制度改革，加工贸易监管从“四方联网”拓展至“八方联网”。组团“走出去”赴南美、俄罗斯、以色列开拓市场、开展招商，赴北京、上海和台湾拜访重点企业。举办新商机推介会，促进镇村空置厂房与客商对接。新引进中集集团、联想增益、世纪互联等重大项目65宗、总投资927亿元。全面减免和规范涉企收费，全面放开再生资源回收市场，尽最大努力帮助企业减轻负担、轻装上阵。

——统筹推进重大产业集聚区规划建设。以新一轮城市总体规划修编为契机，强化主体功能区和经济区规划。水乡特色发展经济区规划通过省审批，被誉为“特色最鲜明的田园型、湿地型生态宝地”。启动10个示范片区、6条特色村落、4段标志性岸线规划建设，总投资486亿元的20个项目顺利推进，实现一年良好开局。松山湖高新区经济高速增长，所有主要经济指标增幅超过20%，台湾高科技园、两岸生物产业基地、中以产业园等建设扎实推进。虎门港与沙田镇统筹发展成效明显。全港集装箱突破190万标箱，货物吞吐量达1.1亿吨，分别从2011年的全省第九、第五跃居第四。生态园获批创建“国家生态工业示范园区”。长安新区基本完成收地1.2万亩，填海有关的各项前期准备工作有序推进。

——加快重大产业项目落地和重要基础设施建设。相继启动两批44个总投资588亿元的重大产业项目。华为终端总部规划设计方案正抓紧报批。粤海项目完成产业规划编制，启动了配套路网建设。加强土地整合，统筹龙湾片区等水乡地块3.6万亩，统筹粤海等重大项目地块3万亩，统筹轨道站点周边土地9600亩，盘活存量土地、处置闲置土地2.2万亩，为重大产业项目落地提供有力支撑。加快47个省市重要基础设施项目建设，完成年度投资184亿元。东莞新火车站正式启用。沿江高速东莞段、水乡大道建成通车。地铁2号线11个站点主体工程封顶，6个区间双线贯通。完成高快速路、国省道和镇村联网路新建改造105公里，建成水利防灾减灾工程30宗、110千伏及以上输变电工程8项、天然气管网110公里。

——推进重大科技平台和投融资平台建设。大学创新城动工建设。北大东莞光电研究院正式奠基。引进清华创新中心、华南设计创新院、岭南创意研究院等院所5家。新增省创新科研团队7家，总数21家，位居全省第三。专利申请和授权量均位居全省第三。成立国内首个检测资源联盟。科技合作周升格为国家级。建立莞商学院。发挥东实集团、水投集团投融资作用，探索城市重大空间和项目的统筹开发。启用民间金融街，推动私募债发展，助力中小微企业融资。引导支持玖龙纸业资产重组，全市主营业务收入超百亿元企业增至7家，超50亿元企业增至33家。

——积极争取先行先试政策红利。主动拜访国家部委和省有关部门，为全市改革发展争取支持。入围国家节能减排财政政策综合示范市，获得每年至少4亿元、3年12亿元以上的中央财政支持。中小河流治理重点县项目获国家1.8亿元补贴，加博会获省财政五年共1.5亿元支持。全国城乡土地生态利用制度综合改革试点获国家批准。入选全国城市共同配送试点城市。获评全国农村社区建设全覆盖示范单位。开展国家外向型经济转型升级综合配套改革试验区申报创建工作，省级可持续发展实验区、清溪保税物流中心（B型）通过省的审批。

——深化商事登记与行政审批制度改革。全面铺开商事登记改革，充分激发了市场活力，新增市场主体同比增长21.5%，市场主体总量达56.7万户，在全省地级市中排名第一。扎实推进后续监管等改革，正式推行网上登记，颁发全国首批电子营业执照。深入推进简政放权，完成第二阶段行政审批事项清理，压减55.1%。积极争取压减158项上级管理审批事项。

——积极扶持镇村基层转型发展。市镇财政安排14.4亿元，用于村（社区）治安、环卫和行政管理支出，减轻了基层负担。32个镇街可支配收入增长24.8%，整体债务率进一步下降。继虎门之后，东城、长安跻身“300亿元俱乐部”。9个欠发达镇生产总值增速全部高于全市平均水平。扎实推进全国农村综合改革示范试点，建成农村集体资产交易和“三资”监管平台，80%以上的村基本完成组级经济统筹。村组两级经营纯收入增长10.6%；资产负债率降至19.3%，降幅为近十年最大。

——扎实推进生态建设和环境治理。开展新一轮绿化大行动，新增绿化面积1.2万亩，完成生态景观林带建设96.4公里。实施南粤水更清行动计划，推进水乡环境五项整治，加强石马河和茅洲河流域治理，基本建成7个环保专业基地。实施清洁空气行动计划，实行市区第三阶段环保限行，淘汰黄标车2.2万辆，更新清洁能源公交车1380辆。环境空气质量优良天数占74.4%。完成市区环保热电厂技改，推广垃圾分类试点。启动万台注塑机示范市创建工作。顺利完成节能减排年度任务。构建大城管格局，试运行数字城管系统。拆除违法建筑49.4万平方米，违建多发态势得到遏制。

——加强社会建设与管理创新。积极创建全省创新社会管理引领区，争当全省社会建设排头兵，强化顶层设计，抓好全省构建现代社会组织体制试点，推动异地务工人员融入东莞。完善政府购买服务机制，转移政府职能87项。打造平安东莞，推进“四化五警”建设，将2万多名治安员整合为辅警。推动警力下沉，提高路面见警率和管事率。深入开展“雷霆扫毒”等行动，严厉打击暴力犯罪和涉众型普遍犯罪。成功蝉联全国社会管理综合治理优秀市。建成劳动关系预警系统，引入社工、律师参与信访调解。扎实推进“两建”工作。设立医疗争议专业调解委员会。抓好食品药品、安全生产、消防安全等专项整治。排查整改突发事件风险隐患627处。社会大局保持和谐稳定。

——切实抓好民生工程和十件实事。投入229.7亿元发展民生事业，向社会承诺的十件实事圆满完成。新建11所公办幼儿园。完成职教城一期建设，理工学校新校、高技能公共实训中心投入使用，技师学院正式开学。公办中职教育实现免费入学。新莞人子女积分入学公办学位增加11.3%。普通高考总录取率等四项高考指标连续两年全省第一。扎实推进文化名城建设，实施百千万文化惠民工程，成功创建首批国家公共文化服务体系示范区。城市文明程度指数位居全国地级市第8名。一批优秀文艺作品获国家级、省级大奖。获得2015年羽毛球苏迪曼杯承办权。建成网球中心和篮球中心。连续第九年提高基本养老金，低保标准提高到每人每月510元。推行居民重大疾病医疗保险，发行600万张新社保卡。新建3386套公租房。为632户低收入家庭提供住房保障。建成一批平价商店、平价医院和诊室，推广平价药包。推行医药分开，取消药品加成。组建市属公立医院管理中心。为8.7万名妇女免费实施“两癌”筛查，为2.6万对夫妇免费提供孕前检查。新莞人与户籍人口享受同等职业技能培训补贴标准。发放就业补贴2.9亿元。妥善应对“3·20”超强龙卷风冰雹灾害、H7N9禽流感疫情。

——全面推进对口帮扶和对外协作等工作。加大市内帮扶力度，81个欠发达村村组两级纯收入增长22%。推进省内“双到”扶贫工作。加大对口帮扶韶关力度，成立专门指挥部，明确7个镇街“一对一”帮扶任务。认真抓好援疆工作，25个援建项目全面竣工。稳步推进援藏工作，8个援建项目超额完成进度。对口援助广西河池、重庆巫山进展顺利。深莞惠产业合作、交通对接等日益加强。穗莞合作不断深化。与呼伦贝尔结为友好城市。国防动员、统计审计、人口计生、外事侨务、工青妇幼、民族宗教、档案方志、科普法普、气象、打私等工作扎实推进。

——着力整治“四风”问题。撤并72.7%的考核检查评比表彰活动，压减47%的节庆、论坛、展会活动。加强对重大政策落实情况、机关作风建设的明查暗访，查摆整改庸懒散奢问题4371个。改进会风文风，厉行勤俭节约，严控各类支出。全面停止新建楼堂馆所，控制和压减镇街财政供养人员。开展整治“小金库”、违规使用专项资金专项行动。推进法治政府建设，公开市级“三公”经费。改革“市民评机关”模式，引入第三方机构测评机关服务质量。59个部门在“阳光热线”与群众进行交流。加快建设网上办事大厅，推动审批事项和服务事项上网办理，不断提升行政效率。

各位代表！回首过去一年，我们砥砺奋进，以高效实干谱写了转型发展的新篇章。这些成绩的取得，离不开上级和市委的坚强领导，离不开市人大、市政协与各民主党派的监督支持，离不开全市广大干部群众、外来投资者、中央和省驻莞单位及驻莞部队的共同努力，离不开港澳台同胞、海外侨胞以及国际友人的关心帮助。在此，我代表市人民政府，表示衷心的感谢！

与此同时，我们也清醒地看到，东莞仍然处在改革攻坚期、发展转型期和矛盾凸显期，有不少问题需要引起高度重视：一是经济平稳较快发展的基础还不牢固。外部环境复杂多变，企业经营压力仍然较大，规模以上工业增加值、进出口等指标增长有所反复。二是区域统筹发展与“三重”建设的力度仍需加大。全市产业发展的层次仍待提升，按主体功能区规划统筹推进的大格局有待完善。重大项目落地动工仍需加速，产能释放尚需时日。三是诸多深层次矛盾有待在深化改革中强力破解。核心竞争力不强、新产业带动不足、资源要素供给紧张等问题仍然存在，企业与市场活力有待进一步激发，扶持企业发展的政策效果仍需加强，重构利益平衡机制难度不小。四是群众关注的热点问题亟待进一步解决。市民对公交出行、人才入户、城市内涝等问题意见较多，治安管理、教育公平、医疗改革等任务艰巨。五是机关作风改进仍然任重道远。“四风”问题仍有待大力改进，财政资金使用绩效有待提升，行政效率、服务水平和管理能力需进一步提高。对这些问题，我们必须认真加以解决。

2014年工作安排

今年是全面贯彻落实党的十八届三中全会精神、全面深化改革的第一年，也是东莞推动高水平崛起的攻坚年。我们应充满信心地看到，我国经济总体处于平稳增长区间，工业化、城镇化快速发展，内需潜力巨大。党的十八届三中全会开启了改革开放新航程，将释放出更多制度红利，对经济社会发展产生重大而深远的影响。我市近年来转型升级积蓄的厚实能量正在逐步释放，创新驱动发展的趋势日益明显，一大批重大产业项目启动建设，如能有效推动这些因素产生叠加效应，东莞就一定能够实现新的更大发展。同时也要清醒地看到，世界经济形势错综复杂，不确定性因素依然很大，欧美债务风险并未完全消除，新的增长动力源尚不明朗，国内生产要素价格持续上涨，我市以制造业为主的工业经济面临更大挑战和压力。今年政府工作的总体要求是：认真贯彻落实党的十八届三中全会、中央经济工作会议、省委十一届三次全会和市委十三届四次全会、市委经济工作会议精神，以提高经济增长质量和效益为中心，按照“稳中求进、稳中求好、稳中求优”的方针，把握“攻坚年”要求，聚力改革攻坚、项目攻坚、效能攻坚，力争稳步迈过“爬坡越坎”的阶段，顺利突破转型升级的“拐点”，努力开拓东莞高水平崛起更加广阔的前景。

具体来讲，改革攻坚，就是要深入贯彻落实上级和市委部署，坚持问题导向，把改革创新贯穿于经济社会发展各个领域各个环节，坚决破除阻碍高水平崛起的瓶颈，增创体制机制新优势，让东莞重新焕发出再领风骚的蓬勃生机。项目攻坚，就是要用好重大平台载体，破解立项审批、征地拆迁等难题，在项目招商、项目落地上求突破，让重大项目对产业转型升级的带动效应尽快显现。效能攻坚，就是要扎实开展群众路线教育实践活动，深入整治“四风”，最大限度促进审批提速、行政提效、服务提质，让全社会感受到市场环境、创业条件、干部作风在一天天好转。

综合考虑各方面因素，今年全市发展的预期目标是：生产总值增长9%，人均生产总值增长7.8%，市公共财政预算收入增长10%，固定资产投资总额增长12%，社会消费品零售总额增长9.5%，外贸出口总额增长5%；服务业增加值占生产总值比重53%，现代服务业增加值占服务业增加值比重58.7%；先进制造业、高技术制造业增加值占规模以上工业增加值比重分别达到45%和35%，研发经费支出占生产总值比重1.94%，发明专利申请量和授权量分别增长15%和25%，全员劳动生产率增长7.8%；单位生产总值能耗下降4.25%，化学需氧量、氨氮、二氧化硫和氮氧化合物等主要污染物排放量分别下降2%、2%、5%和13%；城市居民人均可支配收入、农村居民人均纯收入均增长8%，城镇登记失业率、居民消费价格涨幅分别控制在3%以内和3.5%左右。

围绕以上目标要求，要突出落实好今年市委1号文和市政府1号文精神，坚定不移推进改革发展，脚踏实地促进转型升级。重点做好几方面工作：

一、向改革要动力要红利，增创高水平崛起新优势

十八届三中全会吹响了新一轮全面深化改革的号角，改革再起跑已成为十三亿人民的强烈共识，成为不可阻挡的潮流。已经尝到改革开放甜头的东莞，只有再立潮头，在中央顶层设计的基础上，增强主动改革、自我革命的勇气，打破利益固化的藩篱，激发更加蓬勃的内生动力和改革红利，才能继续在全国城市竞争中处于领先地位，再创“新东莞奇迹”。

以提升开放型经济水平为目标，深化市场取向的经济体制改革。正确处理政府与市场的关系，区别对待不同发展水平与市场发育程度的镇街，因地制宜发挥政府统筹引导作用和市场配置资源的决定性作用。创新加工贸易模式，全面实施加工贸易增效计划，建立开放型经济“四大体系”，创建国家级综合保税区，提升莞港台合作新内涵。放开社会投资限制，探索实行市场准入负面清单的管理模式。搭建民营资本与创新团队、创业项目融合平台，强化市场对科技项目及技术研发的路线选择。以管资本为主加强国有资产监管，对国有企业和国有不动产进行清产核资，引导国有企业逐步退出市场充分竞争行业，以资本运作方式，更多地参与城市基础设施和重大产业平台建设。

以打造商事登记“东莞模式”为突破，推进政府治理体系和治理能力现代化。深化商事登记制度改革，推进电子营业执照应用平台建设，在“宽进”基础上，创新机制倒逼部门加强后置“严管”，为全国市场监管创造新的经验。在其他审批领域试行“宽进严管”。进一步清理压减行政审批事项，力争成为行政审批最少的城市之一。在卫生计生、食品药品监管、文化广电新闻出版等领域探索推进政府职能转变和机构改革。试点在规划、环保、食品药品监管等领域，探索实施按区域设置相关监管部门工作机构。进一步完善市镇财税分成机制，在保持总体稳定的前提下，理顺市镇事权和支出责任。深入推进农村综合改革，加快政务服务中心和综合服务中心建设。提高依法行政水平，推行行政首长出庭应诉、镇街政府法律顾问制度，加强重大政府行为法律审查。继续争取申请成为“较大的市”。

以城乡土地生态利用制度综合改革为抓手，建立健全资源与环境开发保护的科学机制。开展城乡建设用地增减挂钩试点。允许符合条件的集体经营性建设用地出让、租赁、入股。推进农村宅基地制度改革。积极创建国土资源节约集约模范市。创新闲置土地分类处置、地下空间权使用管理等制度。向省争取土地事权审批改革试点。放宽农用地结构调整，有偿易地建设高标准基本农田。高效发展都市农业和都市渔业。完善生态补偿制度，健全环保市场、绿色金融等生态经济激励机制。强化环保责任考核，建立生态环境损害责任终身追究制。加强主体功能区分区生态管理，在市属园区率先探索生态与产业协调发展之路。

以深化户籍制度、公交体制、医药卫生等改革为重点，让社会充满活力又和谐有序。加快入户制度改革，凡符合条件的大专以上应届毕业生、本市职校技校毕业生等10类人才均可直接入户。推进公交体制改革，在市区、跨镇公交和水乡5镇开展试点。深化医药卫生体制改革，着力在健全全民医保体系、完善基本药物制度、推进公立医院改革等方面取得突破。深化文化体制改革，推动文化产业加快发展，提高报业集团、广电传媒集团等经营性文化资产的运作水平。深化物业、停车等民生价格改革。创新社会治理体制，在有效监管前提下加大社会组织培育力度。推进政府购买公共服务制度改革，凡能够购买的，通过合同、委托等方式向社会购买。健全“社工+志愿者”联动机制，充分调动社会力量参与社会治理，进一步增强社会活力。

二、大力扶持实体经济，打造东莞制造升级版

实体经济直接创造物质财富，是社会生产力的集中体现。东莞起步和腾飞靠的是实体经济，高水平崛起同样要大力扶持以先进制造业为核心的实体经济发展，努力让东莞从制造业大市变成制造业强市。

大力发展先进制造业和战略性新兴产业。依托现有产业基础和重大产业平台，集聚发展电气机械、汽车装备、通信设备等先进制造业，大力发展智能终端、云计算、生物医药、新能源、3D打印等战略性新兴产业，培育发展机器人产业。推广物联网智能路灯改造应用。巩固制造业产业优势，进一步延伸和完善产业链，形成上下游产业与制造业互相配套、互相支撑的良好产业生态。促进信息化和工业化融合，加大工业技改资助力度，支持家具、服装等传统优势行业研发自动化生产线，鼓励劳动密集型企业利用机器手等进行智能技术改造。

积极培育制造业企业中的顶梁柱。完善大企业培育政策，深入实施“双百”工程，落实市镇领导挂点服务机制。鼓励大型企业兼并重组，推动企业增资扩产或设立总部。探索建立优质企业镇街间产能扩张及利益共享机制。力争新增主营业务收入超百亿元企业2家、超50亿元企业2家、规模以上企业300家。加力扶持中小微企业，促进产业结构“星月同辉”。

构建政产学研资合作体系。加强科技合作与技术转移。依托散裂中子源项目建设，集聚关联产业和高端实验机构，探索规划建设东莞科学城。推进技术标准体系建设，建立国家模具产品质量监督检验中心。推动大型骨干企业研发机构全覆盖。大力支持企业上市和到“新三板”、区域性股权交易中心挂牌融资。办好民间金融街。推进科技金融创新试验区建设。集聚发展股权投资、小额贷款、融资担保、融资租赁等机构。支持设立民营银行。优化财政扶持方式，提高撬动效果，促使科技和金融创新成为实体经济发展的助推器。

大力发展电子商务和生产性服务业。以获评国家电子商务示范城市为契机，用好市财政每年1.5亿元的专项资金，加大力度扶持电子商务发展。探索实行电商企业集群注册。支持松山湖、专业镇和专业市场建立电商交易平台，深入开展“莞货网上行”等活动。全面启动跨境贸易电子商务服务试点，开展国际邮件业务，让东莞制造产品借助电子商务的翅膀，更多地畅销全省全国和全球市场。大力发展楼宇经济。推进全国城市共同配送试点建设。继续办好加博会、漫博会、台博会、科技合作周等品牌展会。提升物流业发展水平，加快发展工业设计、检测认证、文化创意、信息服务和健康服务等生产性服务业。

营造有利于实体经济发展的“小气候”。抓好现有减负政策落实。通过延续社保缴费费率优惠、下调堤围费征收标准、扩大“营改增”行业范围、减免31项收费等措施，再为企业年度减负22.8亿元以上。完善再生资源回收利用体系，建设再生资源集中处理中心。建立走访企业常态化制度。规范劳动力市场，打击劳务中介不法行为。加快职业教育发展，资助开展技能培训，培养更多技能人才。制定扶持民间资本进入制造业的政策体系，让来东莞发展的企业喜欢东莞、认可东莞、扎根东莞，继续使东莞成为制造业投资的首选之地。

三、实施区域协调发展战略，开启产业地区布局新篇章

加快统筹建设水乡特色发展经济区。全面启动路网基础设施建设，重点推进万望路等8项道路桥梁改扩建工程。启动水乡新城建设，加快万江龙湾等10大示范片区、道滘大罗沙等10大“区中园”的规划建设。突破行政界限，跨镇域整合产业、土地、基础设施和公共服务等资源。实施综合治水、土壤治理、植树造林等行动，重现香飘四季、鱼翔浅底的水乡田园风光。

以大学创新城引领中部地区提升发展水平。加快大学创新城基础设施建设，引进武汉大学、台湾交通大学等创新资源。加快台湾高科技园、两岸生物产业基地、中以产业园建设。对生态园等园区设立严格的产业准入标准，确保投资强度。构建松山湖与周边镇垂直服务、对接联动机制，实现共同发展。东莞暂时还没有高水平的大学，但东莞必须通过非常措施吸引全国乃至全球的高水平大学的资源在东莞生根、开花、结果。

以粤海产业园带动东部各镇壮大产业规模。抓紧确定粤海合作开发模式，加快规划编制和土地统筹，铺开“三纵三横”路网等建设，同步推进招商引资，力争成为省级新区和国家新型城镇化试点。加强山区片等东部各镇产业统筹规划，探索设立高端先进制造业转移园，加强与粤海和深圳的对接，强化产业合作和配套服务，在接受辐射中做强产业经济。

探索打造珠江口东岸现代产业集聚区。加大虎门、长安、沙田、厚街、麻涌和长安新区“五镇一区”区域经济发展统筹力度，摸清岸线、空间和产业等现状，科学研究规划，明确产业定位，主动对接前海、南沙，提升区域发展水平。加快虎门港西大坦1至4号码头泊位报批，推进立沙岛精细化工产业园、麻涌港区粮油加工制造产业园建设。加快长安新区总体规划报批，启动用海规划修编，推进外围海堤立项报批，创新新区控股公司运营管理模式，积极向上争取实施区域围填海。

四、加速重大产业项目落地，燃点经济发展新引擎

再造审批流程，推动项目建设大提速。大刀阔斧推动项目审批流程优化再造，大幅压缩项目审批从立项到施工的平均时间。全面梳理和规范审批办理时限、申报材料等，编制审批目录和流程图，逐步实现全流程电子审批，打造项目建设“高速通道”。凡是其他城市能做到的，东莞一定要做到；其他城市比东莞快的，东莞力争通过改革做得更快一点。

打好重大项目落地攻坚大会战。强化用地保障，新增指标70%用于重大项目建设。发挥东实集团、水投集团等作用，及时协调解决项目融资、征地拆迁等问题，督促在建项目加快进度。增加项目考核权重系数，对先进镇街（园区）给予用地指标奖励。提高项目履约率和开工率，以华为终端项目为龙头，推动年内新启动两批总投资600亿元以上的重大产业项目。

力争重大项目招商取得新成效。建立重大项目招商信息共享、布局统筹、选址流转等利益分享机制，提高镇村统筹招商的积极性。以重大产业平台为载体，开展统筹招商活动，针对欧美日韩台等国家和地区重点招商，主动拜访大型央企民企，力争年内再引进若干个总投资超百亿元及数十亿元的重大项目。

五、实施扩容提质工程，加快新型城镇化进程

中央已就我国城镇化建设作出总体部署。作为城镇化的先行者，东莞必须抓住新的机遇，推动新型城镇化继续走在全省乃至全国前列，积极打造生产空间集约高效、生活空间宜居适度、生态空间山清水秀的美丽东莞，努力形成产业支撑城市发展、城市助推产业升级、产城一体化的良好格局。

巩固发挥城镇化的先发优势，提高城市发展的质量和水平。加强城市顶层设计，加快城市总体规划修编，确保一张蓝图管到底。完善“一中心、多支点”的组团式城镇群格局，不断提高核心城区的首位度，推动各镇加快就地升级发展，打造配套完善、各具特色的城市节点，以现代化的基础设施网络紧密联结，以优美的生态绿化带融洽分隔。结合轨道站点TOD开发，推动城市功能布局优化，进一步完善市镇交通、商贸等功能配套，不断提升城市品质。

推进南城国际商务区建设。按照打造“城市会客厅”的要求，加快南城国际商务区建设，尽快完成土地注入、收地等基础工作，确定准入条件、开发时序与合作模式，抓紧启动市政工程，引进一批总部企业和高端服务业。整合提升中央商圈和各镇商业片区，建设更多环境美、质量好、服务优的商场，让市民愿意在东莞消费，提高消费在国民经济中的拉动作用。

加快轨道交通等重要基础设施建设。力争地铁2号线一、二期土建工程基本完工，实现“隧道通”和“轨道通”，启动运营筹备，为2015年通车做足准备。加强沿线土地统筹开发。推动现代有轨电车试验段规划建设。加快虎门港澳客运码头、高铁虎门站开发建设。加快梨川大桥、东平东江大桥、从莞高速东莞段、环莞快速路二期等建设。扎实推进4G网络、天然气管网、江库联网等工程。加快电网建设，确保投产110千伏及以上输变电工程15项。进一步打通镇村联网路、断头路、跨界路，构建“一张网”全覆盖的交通设施网络。

推动“三旧”改造常态化、长期化。探索组建城市更新和土地整备中心。完善利益平衡机制，引导集体和民营资本参与“三旧”改造。加强规划管控，实施分区控制、差别化地价等政策。每年预留5%新增建设用地指标支持成片更新改造。实行财政补助、不设容积率上限、有条件分割销售等优惠政策，鼓励“工改工”和建设工业大厦，从严控制改建商品房，真正腾挪出新空间来支撑产业转型升级。

以创建活动为抓手，深入推进环境治理和节能减排。积极创建国家生态市、国家森林城市、国家水生态文明城市。完善大岭山、银屏山森林公园二期配套设施，启动常平旗岭等森林公园建设。抓好湿地公园、生态景观林带建设和绿道管理。深入实施南粤水更清、清洁空气等行动计划。加强石马河、茅洲河、沙河、马嘶河、潼湖等跨市河流污染共治，加快挂影洲围中心涌综合整治。试行河长制。有河流污染的镇街至少完成一条内河涌整治，力争实现不黑不臭。完善截污支次管网，提升污水处理厂运营效果。加强东江水源保护，提升供水水质。用好全国节能减排财政政策综合示范市专项资金，推进“绿色水乡”等九大示范工程。加快淘汰黄标车，扩大环保限行区域。划定高污染燃料禁燃区。推动产业配套所需的污染企业进驻环保产业基地，关停不符合环保要求的企业。通过实实在在的举措，让城市融入大自然，让市民望得见蓝天白云、看得见青山绿水、呼吸到清新空气。

六、加大民生投入力度，积极构筑幸福保障网

把建设“平安东莞”作为民生首要任务。保持严打高压态势，重点打击“两抢一盗”、电信诈骗、黄赌毒、传销等违法犯罪活动。加快公安新情报指挥中心平台建设，建成6000个高清视频监控点。落实安全生产“一岗双责”，强化重大隐患排查整改，深化消防安全网格化管理，全面推进消防行政审批与技术审查分离。加快城市综合应急平台建设，强化突发事件联动处置。健全社区矫正工作机制。集中解决积案信访和突出问题，加强欠薪逃匿预警监控，确保社会和谐稳定，进一步提高群众安全感，尽最大努力尽可能让群众走在路上不担心被抢包，住在家里不担心被盗窃，睡在床上不担心有火灾。

认真解决垃圾处理、城市内涝、食品安全等群众关注的热点问题。着力办好十件民生实事。探索垃圾处理利益平衡机制，加快环保热电厂选址、新建和技术改造，建设花园式、公园式、让市民放心的现代垃圾处理厂。加大存量垃圾治理力度，探索将垃圾填埋场改造为休闲绿化公园。推进垃圾分类及收运处置。加强气象灾害预警和市镇内涝整治。加快123宗城乡防灾减灾工程建设。推进通讯基站的规划建设及景观化改造。加快“智慧城市”、“数字城管”建设。积极治理交通拥堵，完成市主干公路交通堵塞点改造工程。开展城市“六乱”和无证医疗机构、生活噪音等专项整治。坚决遏制新增违建，逐步消化存量违建。加强农产品检测和食品药品监管，加大假冒伪劣产品查处力度，确保群众吃得放心、用得安心。

全面发展文教体卫等各项事业。进一步提高公共文化服务水平，支持重大文化项目建设，扶持文艺精品创作。争创全国文明城市三连冠。制定和落实国家历史文化名城保护规划，整合提升虎门销烟遗址、南社和塘尾古村落等旅游资源，推动东莞从旅游客源地向目的地转变，让世界各地不仅认可东莞的经济发展，也被东莞的文化历史所吸引。全面落实创建教育现代化先进市各项措施。提高民办义务教育在校生补助标准。健全民办学校教师待遇政策。大力发展高等教育。推动中职学校专业向工科为主转变。开办外国语学校，异地扩建启智学校，筹办康复实验学校。强化未成年人心理健康教育。完善体育设施。积极筹办苏迪曼杯。办好第八届市运会。推进市人民医院分院、儿童医院、中心血站等建设，开展家庭医生式服务试点。妥善化解医患纠纷，建设平安医院。完善社区卫生服务体系，让群众看病更加方便和便宜。

加强对欠发达镇村、困难群众和对口地区的帮扶。完善市镇主导开发、三级利益共享的统筹发展模式，探索镇村以土地、资金等入股大型基建、园区开发和“三旧”改造。完成村居委会和集体经济组织换届选举。提高市内扶贫实效，力争欠发达村村组两级纯收入增长8%以上、贫困户总脱贫率50%以上。织牢社会保障的安全网，完善社保体系，加大就业资助、教育救助、慈善救助力度。加快深莞惠一体化步伐，深化穗莞战略合作。加强对口帮扶韶关工作，共同成立东韶实业公司，加快“一园一城七组团”开发建设。继续做好省内“双到”扶贫以及对口支援新疆兵团第三师、西藏林芝、广西河池等工作。

七、加强自身建设，打造透明政府、回应政府和公信政府

扎实开展群众路线教育实践活动。严格落实八项规定等要求，深入改进作风，严禁新建楼堂馆所，进一步清理规范庆典、论坛等活动，公开和压减“三公”经费。加强廉政建设，严格落实廉政责任制和廉洁从政各项规定，深入开展“红包”礼金、公款吃喝突出问题专项治理，严肃查办违纪违法案件，主动接受人大、政协、社会舆论等对行政权力的监督。集中解决形式主义、官僚主义、享乐主义和奢靡之风问题，使联系群众成为机关新风尚。畅通利益诉求表达渠道，让市民的诉求有人听、有回应、有结果、有反馈。

建立健全“问题导向、目标管理”的政府工作机制。探索搭建统一投诉平台。通过购买第三方服务开展工作作风和工作绩效明查暗访。深入开展民主评议政风行风。以存在问题和工作目标倒逼整改，促进过程规范、流程再造、程序简化，切实解决落实不力、吃拿卡要等群众、企业和基层反映强烈的突出问题，不断提高政府的执行力，增强群众对政府的信任感。

加强财政资金、集体经济的审计和绩效监管。增强预算完整性和约束力，建立健全政府支出绩效评估考核体系，选取若干村集体试点实施预算制度，强化审计监管，严格监督公共资金依法有效使用。深化农村集体资产交易和“三资”监管平台建设，全面完成村级交易点建设。加强农村廉政风险防控。

加强政府性债务和农村债务控制。坚持量入为出、量力而为、精打细算，杜绝盲目举债搞“政绩工程”，严控新增或续借政府性债务。不能光顾面子，不顾里子，要多做雪中送炭的事，少行锦上添花之举。合理厘清公共基础设施项目投资的市、镇分摊责任。禁止市直部门自行要求镇街增加人员和投入。实行镇街债务风险等级分类管理，重点监控类镇街原则上每年在本级税收分成新增部分至少提取1/3建立偿债准备金。推动村集体增资减债，严禁过度分红甚至借债分红。凡有银信借款的村，在每年积累或土地转让收益中提留一定比例用于还债。

建设全国一流网上办事大厅。完成电子政务云平台二期建设，全面提高网上办理率和网上办事深度，逐步把所有镇街部门平台整合为子平台，把所有资源信息共享到云数据库上。拓展电子审批监察系统，实行网上办事效能监察。通过几年努力，让群众切身感受到“上网一看，一目了然；键盘一按，事情办完”，真正做到少跑路就能办好事、不求人就能办成事。

各位代表！35年前，十一届三中全会为莞邑大地带来了改革开放的春天；35年后，十八届三中全会又一次吹响了全面深化改革的冲锋号。长风破浪会有时，直挂云帆济沧海。让我们在中共东莞市委的坚强领导下，高举改革发展的旗帜，团结和带领全市人民，在爬坡路上攻坚克难，在新征程上锐意前行，为实现伟大的中国梦奋笔书写东莞高水平崛起的新篇章！
